# How digital strategic rhythm influences ambidextrous innovation in high-growth firms: Pathways and boundary conditions

**DOI:** 10.1371/journal.pone.0358573

**Published:** 2026-09-24

**Authors:** Guodong Ye, Ying Ma

**Affiliations:** School of Management, Wuhan University of Technology, Wuhan, Hubei, China; Fudan University, CHINA

## Abstract

High-growth firms must sustain rapid expansion while renewing their innovation capabilities during digitalization. This study examines how digital strategic rhythm, defined as the timing, frequency, and coordination of digital strategic actions, is associated with exploratory and incremental innovation. Drawing on temporal strategy theory, we propose that structural and relational embeddedness provide distinct network mechanisms and that digital sensemaking conditions the conversion of strategic rhythm into network embeddedness. Survey data from 431 middle- and senior-level managers at Chinese high-growth firms were analyzed using confirmatory factor analysis and hierarchical regression. Digital strategic rhythm was positively associated with both forms of innovation. Structural embeddedness partially mediated its association with exploratory innovation, whereas relational embeddedness partially mediated its association with incremental innovation. Digital sensemaking strengthened the association between digital strategic rhythm and innovation outcomes within the structural embeddedness pathway, whereas the corresponding interaction within the relational embeddedness pathway was not significant. These findings extend temporal strategy research by showing how the temporal coordination of digital actions creates distinct network pathways to ambidextrous innovation. They also suggest that high-growth firms should align digital initiatives with network strategies suited to exploratory and incremental innovation.

## 1 Introduction

Over the past decade, digitalization has evolved from a technical issue into a key element of corporate competition and industry governance. It has fundamentally reshaped how firms access resources, collaborate with partners, and reach customers [1,2]. It has also transformed the organization, evaluation, and diffusion of innovation. These changes are particularly pronounced for high‑growth firms (HGFs), typically defined as enterprises whose key indicators, such as revenue, employment, or assets, consistently grow at a substantially higher rate than the industry average [3]. During digitalization, HGFs face a dual challenge: they must exploit existing knowledge to sustain current growth while simultaneously exploring new opportunities in response to digital change [4]. However, their rapid expansion often depends on established routines and existing market knowledge, which facilitate growth but may hinder exploratory innovation [5]. At the same time, digitalization requires firms to develop new capabilities, redesign processes, and actively search for emerging opportunities [6]. Consequently, enabling HGFs to strengthen both incremental and exploratory innovation while maintaining rapid growth and building new capabilities has become a pressing issue.

This tension poses a practical challenge for HGFs. Rapid digital change requires continuous strategic adjustment rather than isolated technology-adoption decisions [2,7]. Yet many HGFs lack the resources and managerial systems needed to coordinate digitalization activities effectively. They therefore require an approach that preserves coherence across transformation initiatives while allowing innovation capabilities to be renewed. Digital strategic rhythm addresses this need by emphasizing the timing, frequency, and coordination of digital strategic actions. It differs from digitalization speed, which concerns how quickly firms adopt technologies; digital strategic rhythm concerns how those actions are sequenced and aligned over time [8]. An appropriate rhythm may help HGFs reconcile short-term growth demands with longer-term innovation needs. Clarifying this process can therefore provide practical guidance for firms seeking sustainable growth in digital environments.

Existing studies have examined digitalization from capability, technology, and organizational perspectives [1,2,9]. They show that innovation depends on firms’ ability to access external knowledge through networks [10,11] and that the temporal pattern of strategic actions influences organizational adaptation and performance [12,13]. However, three gaps remain. First, limited attention has been paid to how firms coordinate repeated digital strategic actions over time or to how this temporal coordination relates to exploratory and incremental innovation. Second, although structural embeddedness provides access to diverse knowledge resources that support exploratory innovation [14], relational embeddedness facilitates trust-based knowledge exchange conducive to incremental innovation [15]. It remains unclear how digital strategic rhythm shapes these network conditions. Third, temporal strategy perspectives emphasize that strategic actions create value when firms can coordinate and interpret them effectively over time [8]. Sensemaking research further explains how organizations understand digitalization and integrate strategic actions [16], while digital sensemaking affects digitalization outcomes through organizational capabilities [17]. However, evidence remains limited on whether digital sensemaking affects structural and relational embeddedness in the same way. Digital sensemaking reflects managers’ ability to interpret digital opportunities and align organizational responses under uncertainty [18]. Examining digital sensemaking therefore helps clarify the boundary conditions under which digital strategic rhythm is translated into different forms of network embeddedness.

To address these gaps, this study examines whether digital strategic rhythm is associated with exploratory and incremental innovation through structural and relational embeddedness and whether digital sensemaking conditions these relationships. It addresses two research questions: (1) How is digital strategic rhythm associated with exploratory and incremental innovation through structural and relational embeddedness? (2) How does digital sensemaking condition the relationships between digital strategic rhythm and these two forms of embeddedness?

## 2 Theoretical framework and hypotheses

Temporal strategy theory argues that the timing, pacing, and synchronization of organizational actions shape how firms adapt to environmental change [8,19]. Whereas traditional strategy perspectives emphasize which actions firms undertake, temporal strategy theory focuses on when those actions occur and how they are coordinated over time [12]. Its central premise is that strategic outcomes depend on both the content and temporal pattern of action [13,20]. Effective temporal coordination can help firms preserve strategic consistency while responding to uncertainty. Existing research, however, has focused mainly on product development, organizational change, and strategic renewal, leaving temporal coordination in digitalization contexts comparatively underexamined. Digitalization differs from conventional strategic change because it requires continuing technological adjustment, organizational adaptation, and ecosystem interaction [2]. Firms therefore need a temporal mechanism that can coordinate digital actions under persistent uncertainty.

Building on temporal strategy theory, we define digital strategic rhythm as the pattern, frequency, and coordination of digital strategic activities over time. The construct captures how firms organize and sequence digitalization initiatives, rather than merely how quickly they adopt digital technologies. Through this rhythm, firms may maintain continuity in transformation, signal strategic commitment, and coordinate interactions with external actors.

Although temporal strategy theory explains how firms coordinate actions over time [21], it does not fully explain how temporal coordination generates innovation outcomes. We extend the theory by integrating network mechanisms. Specifically, we argue that digital strategic rhythm influences innovation through structural and relational embeddedness. Structural embeddedness reflects the network position created through digital strategic activities, whereas relational embeddedness reflects the quality of relationships developed through repeated digital interactions [10,15]. These mechanisms explain how temporal coordination becomes an organizational advantage. Furthermore, temporal strategy theory assumes that firms differ in their ability to interpret and coordinate strategic actions. Digitalization involves ambiguous technological signals and uncertain strategic choices [18]. We therefore introduce digital sensemaking as a boundary condition. By improving the shared interpretation of digital opportunities, digital sensemaking strengthens firms’ ability to translate digital strategic rhythm into advantageous network positions.

This framework extends temporal strategy theory in three respects. First, it conceptualizes digital strategic rhythm as a temporal mechanism specific to digitalization. Second, it identifies network embeddedness as a mechanism linking temporal coordination to innovation outcomes. Third, it specifies digital sensemaking as a boundary condition that may determine whether temporal coordination yields network advantages.

### 2.1 The effects of digital strategic rhythm on ambidextrous innovation

Digitalization has altered how firms organize innovation activities. Because technologies and market requirements change rapidly, firms increasingly need to adjust their digital strategies on an ongoing basis [2,22]. The outcomes of these adjustments depend not only on their content but also on how they are coordinated over time. Temporal strategy research suggests that the timing, frequency, and consistency of strategic actions influence firms’ capacity to adapt and achieve performance outcomes [8,12].

From this perspective, digital strategic rhythm denotes the temporal pattern and coordination of a firm’s digital strategic activities. It captures how the firm schedules digital initiatives, adjusts organizational processes, and preserves strategic consistency throughout digitalization. A stable rhythm can support the continuing allocation of resources, updating of knowledge, and response to environmental change.

Digital strategic rhythm is especially important for HGFs because rapid growth often increases reliance on existing capabilities and established routines. Such reliance may constrain the identification of emerging opportunities and the development of new knowledge domains [5]. A consistent rhythm can encourage the continuing search for external opportunities and the integration of new knowledge, thereby supporting exploratory innovation. This form of innovation requires firms to move beyond established knowledge boundaries and develop novel technologies, products, or solutions [4].

Digital strategic rhythm may also support incremental innovation by sustaining processes of continuous improvement. Incremental innovation refines existing products, processes, and technologies through repeated adjustments [23]. A coordinated rhythm can help firms respond to customer feedback, optimize operations, and strengthen existing capabilities. Digital strategic rhythm may therefore provide a temporal foundation for both exploratory and incremental innovation. On this basis, we propose:

**H1a:** *Digital strategic rhythm positively influences exploratory innovation.*

**H1b:** *Digital strategic rhythm positively influences incremental innovation.*

### 2.2 The mediating role of digital network embeddedness

Digital strategic rhythm alone does not ensure innovation; firms must convert digital actions into valuable external resources. Such conversion often requires collaboration because digital innovation involves knowledge exchange, technological integration, and resource coordination across organizational boundaries [9,24]. Digital network embeddedness therefore offers a mechanism linking digital strategic rhythm to ambidextrous innovation. It describes the extent to which firms are integrated into digital innovation networks through their network positions and inter-organizational relationships. The construct comprises structural and relational embeddedness [10,15]. Structural embeddedness reflects a firm’s network position and access to diverse knowledge resources [14], whereas relational embeddedness reflects the quality of relationships among network actors and the effectiveness of knowledge exchange [25].

Digital strategic rhythm may strengthen structural embeddedness because continuing digital activity creates repeated opportunities to interact with diverse external actors. A consistent rhythm also signals the firm’s digital commitment and intentions for future collaboration, increasing its visibility and facilitating broader network connections. Stronger structural embeddedness, in turn, gives firms access to heterogeneous knowledge and opportunities beyond existing technological boundaries. This access is particularly relevant to exploratory innovation, which depends on searching for, recombining, and integrating unfamiliar information [4,26]. Firms in advantageous network positions can draw on diverse knowledge domains and combine their elements to develop novel solutions.

Digital strategic rhythm may also strengthen relational embeddedness by sustaining interaction with key partners. Digitalization commonly requires close coordination around shared data, integrated systems, and joint problem-solving. Repeated interaction can foster trust, shared expectations, and routines for cooperation [15]. These relational resources are relevant to incremental innovation because continuous improvement depends on efficient knowledge transfer and coordination among existing partners [23,27]. Digital network embeddedness may therefore connect digital strategic rhythm to distinct innovation outcomes. Specifically:

**H2a:** *Structural embeddedness mediates the relationship between digital strategic rhythm and exploratory innovation.*

**H2b:** *Relational embeddedness mediates the relationship between digital strategic rhythm and incremental innovation.*

### 2.3 The moderating role of digital sensemaking

Drawing on organizational sensemaking theory, we propose that digital sensemaking—the social process through which an organization collectively interprets digital technologies and innovation contexts to form shared cognition [28]—positively moderates the relationship between digital strategic rhythm and structural network embeddedness. Because digital innovation is open, editable, and involves multiple actors [29], organizations require ongoing sensemaking to align internal views of technology and strategy [30] and coordinate external actions. This moderating role operates through three mechanisms. First, strong digital sensemaking enables a firm to articulate the purpose and logic of its digital strategy adjustments to external network actors more clearly and consistently, making its strategic signals more credible and interpretable [31]. This clarity reduces ambiguity, allows partners to align their expectations with the firm’s strategic direction, and accelerates network formation and partner attraction. Second, strong sensemaking fosters emotional consensus and cognitive alignment regarding technology and innovation goals within and beyond the firm [32]. It thereby reduces collaborative friction and facilitates network expansion. Third, such cognitive alignment improves the firm’s ability to identify and bridge knowledge domains and thus to occupy and maintain structural holes that provide access to diverse information and resources [14]. By contrast, weak digital sensemaking produces ambiguous strategic signals and cognitive divergence among actors [33]. External partners may then be unable to interpret the firm’s intentions reliably or commit to collaboration, weakening the association between digital strategic rhythm and network structure. Digital sensemaking therefore provides a cognitive foundation for converting strategic dynamism into structural network advantages. Hence:

**H3a:** *Digital sensemaking positively moderates the effect of digital strategic rhythm on innovation outcomes through the structural embeddedness pathway.*

Similarly, digital sensemaking—as a social process of collectively interpreting digital technologies, strategic intentions, and collaborative contexts to develop shared cognition [34]—may strengthen the association of digital strategic rhythm with relationship strength and quality. When digital sensemaking is strong, the organization develops a coherent interpretation of the logic, goals, and technological pathways of its digital strategy [35]. This internal cognitive unity and clarity enable the firm to communicate its strategic commitments and expectations of cooperation to external partners more consistently and credibly [36]. Doing so accelerates trust building and the formation of reciprocal norms, thereby improving relationship quality. A clear shared understanding also lowers cognitive friction and interpretation costs in cross‑organizational collaboration. Both parties can then focus on deepening the integration of data, processes, and systems, thereby increasing the frequency and depth of interaction. When digital sensemaking is weak, internal interpretations of digital strategy may diverge or remain vague, producing confused or contradictory external signals [37]. This ambiguity not only impedes trust building and improvements in relationship quality but also makes cross‑organizational digital collaboration less efficient, thereby preventing strategic rhythm from being translated into deeper relationships. Thus:

**H3b:** *Digital sensemaking positively moderates the effect of digital strategic rhythm on innovation outcomes through the relational embeddedness pathway.*

**Fig 1:**
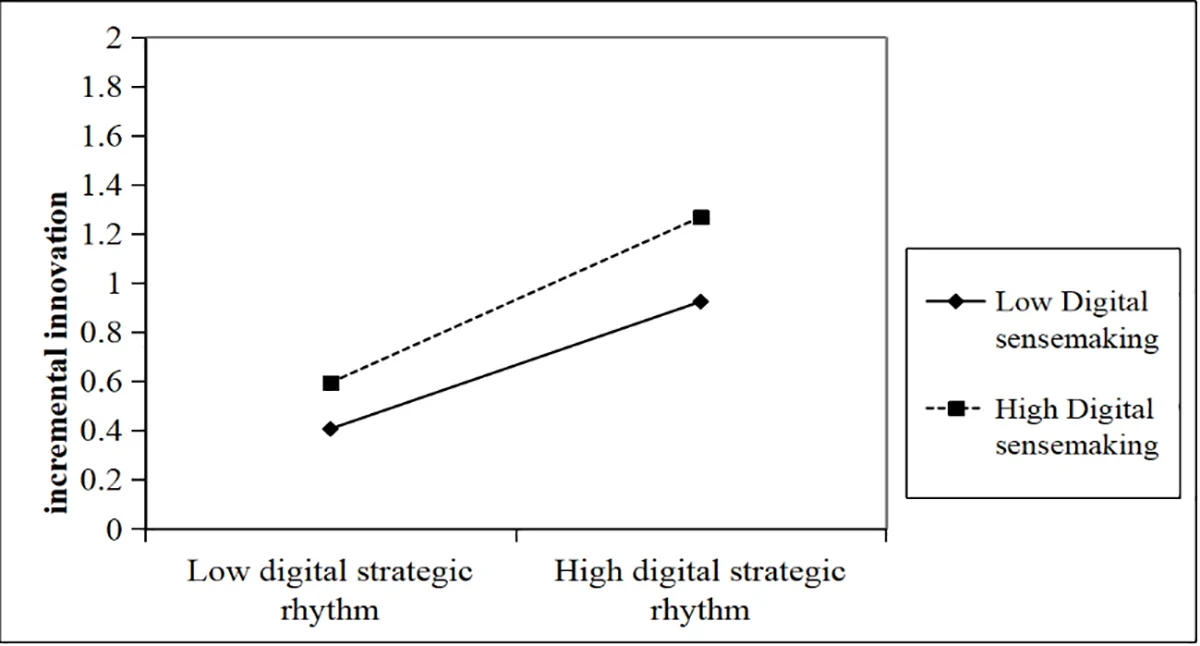
Empirical model of digital network embeddedness paths.

The resulting conceptual model is presented in Fig 1.

## 3 Research design

### 3.1 Sample

We surveyed middle- and senior-level managers at high‑growth firms in Beijing, Shanghai, Shandong, and Jiangsu. Drawing on *the Classification Guide for High‑Growth Enterprises* issued by the relevant Chinese authorities, we identified high‑growth firms using two criteria: an average annual revenue growth rate exceeding 20% over the previous three years and an average R&D investment intensity exceeding 2.5% of total revenue over the same period. We used two procedural measures to ensure that respondents could represent their firms’ strategic orientations. First, we deliberately targeted middle- and senior-level managers who were directly involved in or had comprehensive knowledge of their firms’ digital strategy formulation and external partnership decisions. Second, before administering the formal questionnaire, we included a screening item that assessed respondents’ familiarity with their firm’s digital strategy and network collaborations on a five-point Likert scale. Only respondents scoring 4 or 5 were retained, increasing the likelihood that the final sample comprised reliable key informants.

Data were collected using paper-based and online questionnaires. We followed several procedures. First, the items were developed carefully. A doctoral student in English translated the English-language questionnaire into Chinese, after which a doctoral student in management reviewed and refined the translated items. We also revised items following interviews with managers, including by emphasizing digitalization, and adapted the wording to conventional Chinese usage (e.g., by replacing “organization” with “company”). Second, questionnaires were obtained from three sources: (1) through our research team’s network, we contacted chief executive officers of several HGFs, obtained access, and assisted with questionnaire distribution and collection; (2) during breaks at a management training conference, we distributed questionnaires to HGF managers; and (3) we sent electronic questionnaires through an online survey platform to managers who could not attend the conference or were referred through personal contacts. Third, before distribution, we explained the study’s purpose, the anonymity of responses, and the completion instructions. In the relational embeddedness section, we included two consistency-check items, “Our company communicates truthfully with digital partners” and “Our company can moderately lie when communicating with digital partners”, separated by nine other items. Questionnaires were excluded when the same response was given to both items.

Of the 632 questionnaires distributed, 431 remained after questionnaires with missing responses, multiple responses, or failed consistency checks were excluded, yielding a valid-response rate of 68.2%. To assess non‑response bias, we compared the on‑site and online subsamples with respect to industry and other respondent characteristics. No significant differences were detected, suggesting that non‑response bias was unlikely to be substantial. We assessed potential common method bias using Harman’s single‑factor test on all self‑reported variables, based on an unrotated principal component analysis retaining factors with eigenvalues greater than 1. The first factor accounted for 16.125% of the total variance, well below the recommended threshold of 50%. The cumulative variance explained by all factors was 65.713%, exceeding the 60% benchmark. These results suggest that common method bias was unlikely to pose a serious threat to the validity of the findings [38].

The final sample had the following characteristics. Firm age: 1–2 years (7%), 3–5 years (42.5%), 6–10 years (38.3%), and >10 years (12.3%). Industry: electronics/telecommunications (6%), mechanical manufacturing (17.6%), biomedicine (16.2%), software services (53.1%), and other industries (7%). Ownership: joint venture (10%), private (40.8%), and state‑owned (49.2%). Firm size: ≤ 50 employees (37.1%), 51–200 employees (44.3%), 201–500 employees (10.7%), 501–1,000 employees (6.5%), and >1,000 employees (1.4%). Market share relative to competitors: much smaller (11.6%), smaller (4.9%), approximately the same (77.3%), larger (1.9%), and much larger (4.9%).

### 3.2 Measurement

We used established, well‑tested scales with minor adaptations. All items were rated on a five-point Likert scale (1 = strongly disagree; 5 = strongly agree).

Digital strategic rhythm was measured using five items adapted from [39]. An example item was: “Our firm regularly applies digital technologies to launch new products/services, start new businesses, or enter new markets, according to a set schedule.”

Network embeddedness was measured using a scale adapted from [40]. Structural embeddedness (four items) captured network position and centrality; an example item was: “Upstream and downstream firms often establish contact with digital native firms through our company.” Relational embeddedness (six items) captured the closeness of interactions with digital partners; an example item was: “Our company and our digital partners always keep their promises.”

Ambidextrous innovation was measured using six exploratory innovation items from [41] and six incremental innovation items from [42]. Example items were “Inventing new products and services” for exploratory innovation and “Improving the supply of existing products and services” for incremental innovation.

Digital sensemaking was measured using a five-item scale adapted from [43] that focuses on retrospective sensemaking in digital innovation. An example item was: “If something goes wrong in digital technology innovation, we try hard to find the cause.” We selected this scale because its reliability and validity have been established and it was published in Industrial Marketing Management, a reputable journal.

The control variables were firm age, industry, ownership, firm size, and market share; all were categorical.

## 4 Results

### 4.1 Measurement model

Before testing the hypothesized relationships, we evaluated the measurement properties of each construct (Table 1). Cronbach’s alpha values ranged from 0.839 to 0.919, and composite reliability values ranged from 0.886 to 0.940. All values exceeded the recommended threshold of 0.70, indicating satisfactory internal consistency. Average variance extracted ranged from 0.610 to 0.758 and exceeded the recommended threshold of 0.50 [44], indicating that each construct accounted for more than 50% of the variance in its indicators. Overall, Table 1 provides evidence of satisfactory indicator reliability, internal consistency, and convergent validity.

**Table 1:** Reliability.

Latent Variable	Item	Factor Loading	CR	AVE	Cronbach’s α
Digital Strategic Rhythm	DSR1	0.895	0.940	0.758	0.919
	DSR2	0.901			
	DSR3	0.880			
	DSR4	0.804			
	DSR5	0.869			
Structural Embeddedness	SE1	0.817	0.912	0.721	0.869
	SE2	0.884			
	SE3	0.868			
	SE4	0.825			
Relational Embeddedness	RE1	0.864	0.912	0.635	0.882
	RE2	0.849			
	RE3	0.839			
	RE4	0.849			
	RE5	0.648			
	RE6	0.705			
Digital Sensemaking	DS1	0.763	0.886	0.610	0.839
	DS2	0.840			
	DS3	0.756			
	DS4	0.823			
	DS5	0.715			
Exploratory Innovation	EI1	0.750	0.909	0.625	0.880
	EI2	0.833			
	EI3	0.807			
	EI4	0.794			
	EI5	0.781			
	EI6	0.776			
Incremental Innovation	II1	0.818	0.916	0.644	0.889
	II2	0.818			
	II3	0.826			
	II4	0.782			
	II5	0.792			
	II6	0.778			

**Note:** DSR = digital strategic rhythm, SE = structural embeddedness, RE = relational embeddedness, DS = digital sensemaking, EI = exploratory innovation, and II = incremental innovation.

Table 2 reports descriptive statistics, correlations, and evidence of discriminant validity for the study variables. The construct means ranged from 3.26 to 4.01, and the standard deviations ranged from 0.69 to 0.83. Interconstruct correlations ranged from 0.215 to 0.783. We assessed discriminant validity using the Fornell–Larcker criterion. The square roots of the average variance extracted, shown on the diagonal, ranged from 0.781 to 0.871 and exceeded the corresponding interconstruct correlations. These results indicate acceptable discriminant validity [44].

**Table 2:** Discriminant validity.

Construct	Mean	SD	DSR	SE	RE	DS	EI	II
DSR	3.91	0.83	**0.871**					
SE	3.79	0.79	0.559	**0.849**				
RE	3.83	0.71	0.633	0.783	**0.797**			
DS	3.26	0.81	0.215	0.387	0.384	**0.781**		
EI	4.01	0.71	0.713	0.588	0.688	0.239	**0.791**	
II	3.77	0.69	0.609	0.630	0.666	0.373	0.730	**0.802**

**Note:** DSR = digital strategic rhythm, SE = structural embeddedness, RE = relational embeddedness, DS = digital sensemaking, EI = exploratory innovation, and II = incremental innovation.

### 4.2 Structural model

Confirmatory factor analysis was conducted to assess the distinctiveness of the six focal constructs. As shown in Table 3, the hypothesized six-factor model fit the data acceptably: χ^2^(449) = 1442.92, χ^2^/df = 3.214, RMSEA = 0.072, SRMR = 0.061, NFI = 0.96, and CFI = 0.98. The χ^2^/df value was below 5.00; the RMSEA and SRMR values were below 0.08; and the NFI and CFI values exceeded 0.90, indicating acceptable measurement-model fit [45]. Moreover, the six-factor model fit the data substantially better than the five-, four-, three-, two-, and single-factor models [46,47]. These results support the proposed six-factor structure and indicate that the six constructs are empirically distinguishable.

**Table 3:** Confirmatory factor analysis of variables.

Model	χ^2^	df	χ^2^/df	RMSEA	SRMR	NFI	CFI
Six-factor model	1442.92	449	3.214	0.072	0.061	0.96	0.98
Five-factor model	2767.37	454	6.096	0.109	0.094	0.94	0.96
Four-factor model	3215.08	458	7.020	0.118	0.095	0.94	0.95
Three-factor model	3348.97	461	7.265	0.121	0.095	0.94	0.95
Two-factor model	4198.91	463	9.069	0.137	0.100	0.92	0.93
Single-factor model	4943.02	465	10.630	0.150	0.110	0.91	0.92

### 4.3 Hypothesis testing

We used hierarchical regression analysis to test the hypotheses. Firm age, industry, ownership, firm size, and market share were entered as control variables. The maximum variance inflation factor across the reported models was 1.963, indicating that multicollinearity was not a material concern. The models are presented according to the hypotheses tested. Coefficients are reported as unstandardized estimates (B), with standard errors in parentheses.

#### 4.3.1 Main effects.

H1a predicted a positive association between digital strategic rhythm and exploratory innovation, whereas H1b predicted a positive association between digital strategic rhythm and incremental innovation. As shown in Table 4, digital strategic rhythm was positively associated with exploratory innovation (B = 0.574, SE = 0.029, p < 0.01) and incremental innovation (B = 0.492, SE = 0.033, p < 0.01). The respective models explained 54.2% and 38.8% of the variance in these outcomes. These results supported H1a and H1b.

**Table 4:** Main effects of digital strategic rhythm on innovation.

Variable	M1 (Exploratory Innovation)	M2 (Incremental Innovation)
Intercept	0.923^**^ (0.188)	1.336^**^ (0.211)
Firm age	0.013 (0.031)	0.062 (0.035)
Industry	0.104^**^ (0.025)	0.016 (0.028)
Ownership	−0.002 (0.037)	0.013 (0.041)
Firm size	0.074^**^ (0.026)	0.054 (0.029)
Market share	0.113^**^ (0.030)	0.059 (0.034)
Digital strategic rhythm	0.574^**^ (0.029)	0.492^**^ (0.033)
R^2^	0.542	0.388
Adjusted R^2^	0.535	0.379
F	83.502^**^	44.739^**^
Maximum VIF	1.214	1.214

**Note:** * p < 0.05; ** p < 0.01. Entries are B coefficients with standard errors in parentheses.

#### 4.3.2 Mediation effects.

H2a proposed that structural embeddedness would mediate the relationship between digital strategic rhythm and exploratory innovation. Digital strategic rhythm positively predicted structural embeddedness (B = 0.511, SE = 0.039, p < 0.01). When structural embeddedness was added to the exploratory innovation model, it had a positive coefficient (B = 0.237, SE = 0.035, p < 0.01), while the coefficient for digital strategic rhythm decreased from 0.574 to 0.452 but remained significant. The product of the two coefficients was approximately 0.121, a pattern consistent with partial mediation.

H2b proposed that relational embeddedness would mediate the relationship between digital strategic rhythm and incremental innovation. Digital strategic rhythm positively predicted relational embeddedness (B = 0.515, SE = 0.033, p < 0.01). When relational embeddedness was added to the incremental innovation model, it had a positive coefficient (B = 0.441, SE = 0.044, p < 0.01), while the coefficient for digital strategic rhythm decreased from 0.492 to 0.265 but remained significant. The product of the two coefficients was approximately 0.227, a pattern consistent with partial mediation.

Accordingly, the regression results show patterns consistent with partial mediation for both H2a and H2b. Digital strategic rhythm is associated with exploratory innovation partly through structural embeddedness and with incremental innovation partly through relational embeddedness (Table 5).

**Table 5:** Regression results for the mediation models.

Variable	M3 (SE)	M4 (EI + SE)	M5 (RE)	M6 (II + RE)
Intercept	1.002^**^ (0.251)	0.686^**^ (0.182)	1.068^**^ (0.211)	0.865^**^ (0.195)
Firm age	0.126^**^ (0.042)	−0.017 (0.030)	0.093^**^ (0.035)	0.021 (0.032)
Industry	0.010 (0.033)	0.102^**^ (0.023)	0.058^*^ (0.028)	−0.010 (0.025)
Ownership	0.083 (0.049)	−0.022 (0.035)	0.020 (0.041)	0.004 (0.037)
Firm size	0.063 (0.035)	0.059^*^ (0.025)	0.054 (0.029)	0.030 (0.027)
Market share	0.039 (0.040)	0.104^**^ (0.029)	0.058 (0.034)	0.033 (0.031)
Digital strategic rhythm	0.511^**^ (0.039)	0.452^**^ (0.033)	0.515^**^ (0.033)	0.265^**^ (0.037)
Structural embeddedness	—	0.237^**^ (0.035)	—	—
Relational embeddedness	—	—	—	0.441^**^ (0.044)
R^2^	0.348	0.587	0.428	0.506
Adjusted R^2^	0.339	0.581	0.420	0.498
ΔR^2^	0.262	0.045	0.328	0.118
F	37.681^**^	86.045^**^	52.964^**^	61.988^**^
Maximum VIF	1.214	1.533	1.214	1.749

**Note:** * p < 0.05; ** p < 0.01. Entries are B coefficients with standard errors in parentheses. SE = structural embeddedness; RE = relational embeddedness; EI = exploratory innovation; II = incremental innovation. ΔR^2^ for M4 and M6 was recalculated relative to the corresponding main-effect model.

#### 4.3.3 Moderating effects.

H3a proposed that digital sensemaking positively moderates the effect of digital strategic rhythm on innovation outcomes through the structural embeddedness pathway. As shown in Table 6, the interaction between digital strategic rhythm and digital sensemaking did not significantly predict structural embeddedness in M7 (B = 0.002, non-significant). However, when structural embeddedness was included in the outcome models, the interaction term was positive and significant for exploratory innovation in M9 (B = 0.038, p < 0.05) and incremental innovation in M11 (B = 0.039, p < 0.05). These findings were interpreted as indicating that digital sensemaking strengthens the association between digital strategic rhythm and innovation outcomes in models that include structural embeddedness. H3a was therefore considered supported.

**Table 6:** Regression results for the moderation models.

Variable	M7 (SE)	M8 (RE)	M9 (EI)	M10 (EI)	M11 (II)	M12 (II)
Intercept	0.463 (0.252)	0.650^**^ (0.211)	0.707^**^ (0.189)	0.574^**^ (0.183)	0.798^**^ (0.199)	0.695^**^ (0.199)
Firm age	0.115^**^ (0.040)	0.081^*^ (0.033)	−0.020 (0.030)	−0.023 (0.029)	0.014 (0.032)	0.019 (0.031)
Industry	0.012 (0.031)	0.056^*^ (0.026)	0.099^**^ (0.023)	0.081^**^ (0.023)	0.011 (0.025)	−0.007 (0.025)
Ownership	0.078 (0.047)	0.021 (0.039)	−0.016 (0.035)	−0.006 (0.034)	−0.010 (0.037)	0.006 (0.037)
Firm size	0.037 (0.033)	0.033 (0.028)	0.059^*^ (0.025)	0.056^*^ (0.024)	0.023 (0.026)	0.022 (0.026)
Market share	0.034 (0.038)	0.053 (0.032)	0.103^**^ (0.029)	0.091^**^ (0.028)	0.043 (0.030)	0.034 (0.030)
DSR	0.458^**^ (0.039)	0.460^**^ (0.032)	0.442^**^ (0.033)	0.379^**^ (0.034)	0.298^**^ (0.035)	0.266^**^ (0.037)
DS	0.258^**^ (0.038)	0.224^**^ (0.032)	0.015 (0.030)	−0.008 (0.029)	0.133^**^ (0.031)	0.128^**^ (0.031)
DSR × DS	0.002 (0.024)	0.040 (0.020)	0.038^*^ (0.018)	0.024 (0.018)	0.039^*^ (0.019)	0.024 (0.019)
SE			0.233^**^ (0.036)		0.308^**^ (0.038)	
RE				0.371^**^ (0.042)		0.379^**^ (0.045)
R^2^	0.413	0.490	0.592	0.623	0.522	0.526
R^2^_adj_	0.402	0.481	0.583	0.615	0.511	0.516
*ΔR* ^*2*^	0.413	0.490	0.592	0.623	0.522	0.526
F	37.096^**^	50.772^**^	67.815^**^	77.189^**^	51.004^**^	51.865^**^
Maximum VIF	1.218	1.218	1.703	1.963	1.703	1.963

**Note:** * p < 0.05; ** p < 0.01. Entries are B coefficients with standard errors in parentheses. DSR = digital strategic rhythm; DS = digital sensemaking.

H3b proposed that digital sensemaking positively moderates the effect of digital strategic rhythm on innovation outcomes through the relational embeddedness pathway. The interaction between digital strategic rhythm and digital sensemaking did not significantly predict relational embeddedness in M8 (B = 0.040, non-significant). When relational embeddedness was included in the outcome models, the interaction term remained non-significant for exploratory innovation in M10 (B = 0.024) and incremental innovation in M12 (B = 0.024). Thus, the results did not indicate that digital sensemaking strengthened the association between digital strategic rhythm and innovation outcomes in models that included relational embeddedness. H3b was therefore not supported.

To illustrate the moderating relationships more clearly [48], we plotted the associations between digital strategic rhythm and each innovation outcome at one standard deviation above and below the mean of digital sensemaking.

**Fig 2:**
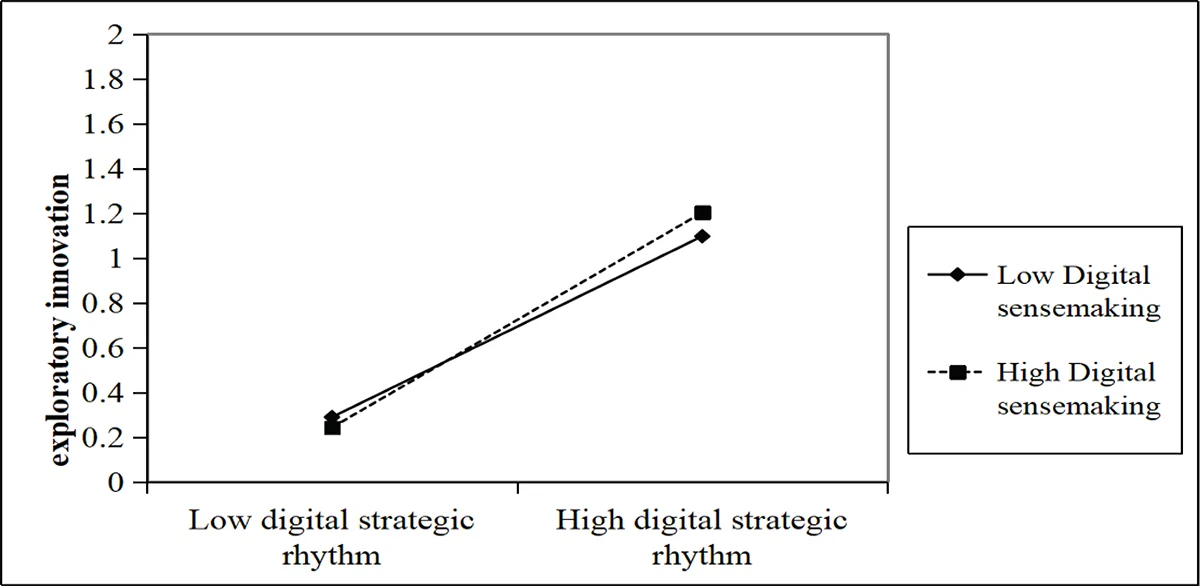
Moderating role of digital sensemaking in the relationship between digital strategic rhythm and exploratory innovation in the structural embeddedness model.

Fig 2 plots exploratory innovation against digital strategic rhythm at low and high levels of digital sensemaking. When digital strategic rhythm is low, predicted exploratory innovation is low under both conditions, with the low-sensemaking line slightly above the high-sensemaking line. As digital strategic rhythm increases, exploratory innovation increases under both conditions. At high digital strategic rhythm, predicted exploratory innovation is higher under high than under low digital sensemaking, and the gap between the lines widens. Both lines therefore slope upwards, with a slightly steeper slope under high digital sensemaking. This pattern is consistent with positive moderation: digital sensemaking has little apparent benefit at low digital strategic rhythm but strengthens the association at high digital strategic rhythm.

This pattern can be understood through three complementary mechanisms involving digital strategic rhythm, digital sensemaking, and exploratory innovation. First, digital strategic rhythm may improve a firm’s response to new technologies and market opportunities by increasing the frequency of trial‑and‑error and accelerating resource reconfiguration. Because exploratory innovation relies on uncertain knowledge and cross‑boundary experimentation, a stronger rhythm may reduce delays and allow firms to act within relevant technological windows. Second, digital sensemaking creates a shared understanding of digital goals and technological value, reducing cognitive inconsistency and fragmentation in exploratory activities. Without shared cognition, repeated innovation experiments may become fragmented or follow conflicting paths. Stronger sensemaking makes strategic signals more interpretable and gives exploratory activities greater organizational coherence. Third, a stronger digital strategic rhythm is often accompanied by more frequent external interactions and knowledge flows. Digital sensemaking helps firms absorb this external knowledge and recombine it across boundaries, which may explain why predicted exploratory innovation is highest when both digital strategic rhythm and digital sensemaking are high.

**Fig 3:**
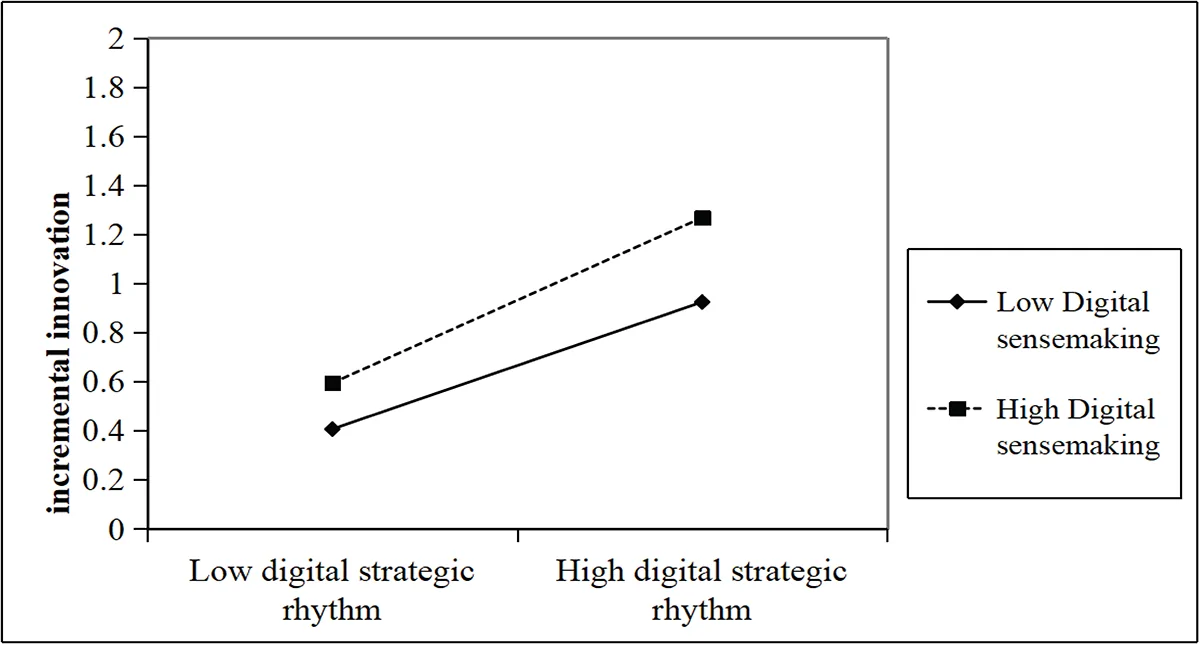
Moderating role of digital sensemaking in the relationship between digital strategic rhythm and incremental innovation in the structural embeddedness model.

Fig 3 plots incremental innovation against digital strategic rhythm at low and high levels of digital sensemaking. When digital strategic rhythm is low, predicted incremental innovation is moderately low, although the high-sensemaking line lies above the low-sensemaking line. This pattern suggests that a shared digital understanding may support incremental innovation, even when the rhythm of digital strategy implementation is limited, by facilitating knowledge integration and process optimization. As digital strategic rhythm increases, predicted incremental innovation increases under both conditions. At high digital strategic rhythm, the predicted level remains higher under high digital sensemaking, and the gap between the lines widens. Both lines therefore slope upwards, with the high-sensemaking line displaying the steeper slope. This pattern is consistent with positive moderation of the association between digital strategic rhythm and incremental innovation.

This pattern can be understood through the support that digital strategic rhythm provides for exploitation‑oriented innovation and the complementary role of digital sensemaking. First, incremental innovation relies on the recombination of existing knowledge, process optimization, and capability development. It therefore depends more heavily than exploratory innovation on stable resource inputs and continuous improvement. By increasing the frequency of system updates, the pace of process iteration, and the use of data, a stronger digital strategic rhythm may help firms optimize existing operations continuously. Second, digital sensemaking develops a shared understanding of digital goals, data value, and technological applications. This shared understanding reduces cross‑departmental coordination friction and obstacles to knowledge orchestration, thereby improving the integration of knowledge and processes. It may also explain why predicted incremental innovation remains relatively high under strong digital sensemaking when digital strategic rhythm is low. Third, frequent system adjustments and business reconfigurations may create fatigue and conflicting paths when shared cognition is absent. Digital sensemaking helps firms maintain a stable direction for improvement during frequent strategic adjustments, which may explain the widening gap between the two lines at high digital strategic rhythm.

## 5 Discussion

This study addressed two research questions: how digital strategic rhythm is related to exploratory and incremental innovation through digital network embeddedness, and how digital sensemaking conditions these relationships. The findings indicate that digital strategic rhythm is positively associated with both forms of innovation through distinct network mechanisms. Structural embeddedness accounts for part of its association with exploratory innovation, whereas relational embeddedness accounts for part of its association with incremental innovation. These findings extend temporal strategy research by showing that the temporal coordination of digital actions is linked to external network advantages. They therefore extend prior work that has primarily emphasized the timing and synchronization of strategic actions in organizational adaptation [8,12].

The two network mechanisms appear to operate differently. Exploratory innovation requires firms to access heterogeneous knowledge and search beyond existing knowledge domains [4]. A stable digital strategic rhythm increases interaction with diverse external actors, while structural embeddedness provides access to non-redundant knowledge and emerging opportunities [10,14]. By contrast, incremental innovation depends more on continuous improvement and coordination with existing partners [23]. Digital strategic rhythm may strengthen relational embeddedness through repeated interaction and stable cooperation, facilitating knowledge exchange by building trust and shared expectations [15,25]. These findings clarify how the same temporal pattern of digital actions may be associated with different innovation outcomes through distinct forms of network embeddedness.

Regarding the second research question, digital sensemaking strengthened the association between digital strategic rhythm and innovation outcomes in the structural embeddedness models, whereas the corresponding interaction was not significant in the relational embeddedness models. Prior research emphasizes that digitalization requires firms to interpret technological change and develop a shared understanding [18]. The findings refine this view by suggesting that the role of digital sensemaking differs across network contexts. Structural embeddedness provides access to diverse knowledge and new network opportunities, which may make this innovation pathway more responsive to sensemaking. By contrast, relational embeddedness depends more strongly on repeated interaction, accumulated trust, and relationship history [15], which may explain the weaker moderating result in the relational models.

### 5.1 Theoretical contributions

This study makes three theoretical contributions. First, it extends temporal strategy research to the context of digitalization. Prior research shows that the timing and coordination of strategic actions influence organizational adaptation and performance [8,12], but offers limited explanation of how temporal coordination operates when firms repeatedly adjust digital strategies. By introducing digital strategic rhythm and demonstrating its associations with exploratory and incremental innovation, this study identifies the timing, frequency, and coordination of digital strategic actions as an important temporal mechanism of innovation. It thereby extends temporal strategy research from general patterns of strategic timing to the temporal organization of digital strategic action.

Second, this study extends network embeddedness research by explaining how temporally coordinated strategic actions may shape network conditions that are subsequently associated with innovation outcomes. Previous network research has primarily examined how external relationships provide firms with knowledge and strategic resources [10]. Structural embeddedness is associated with access to diverse knowledge [14], whereas relational embeddedness facilitates trust-based knowledge exchange [15]. Our findings move beyond treating these network conditions solely as antecedents of innovation by showing that they may also develop from the temporal coordination of digital strategic actions. Specifically, digital strategic rhythm was associated with exploratory innovation partly through structural embeddedness and with incremental innovation partly through relational embeddedness. This distinction clarifies how the same temporal pattern of digital actions can be linked to different innovation outcomes through distinct network mechanisms.

Third, this study refines digital sensemaking research by identifying a differentiated boundary role across network mechanisms. Existing research shows that sensemaking helps organizations interpret digital change and coordinate organizational responses [16,17], but provides limited evidence on whether this capability contributes equally to different forms of network development. Our findings indicate that digital sensemaking strengthens the association involving structural embeddedness, whereas its role in the relational embeddedness models is limited. This asymmetric pattern suggests that digital sensemaking may be particularly valuable when firms seek to identify and develop new network positions, while relationship development depends more strongly on repeated interaction and accumulated trust. The findings therefore refine current understanding by showing that the relevance of digital sensemaking may depend on the type of network mechanism.

### 5.2 Practical implications

This study has several practical implications for managers of HGFs. First, digitalization should be managed as an ongoing process of temporal coordination rather than as a series of isolated technology investments. Prior research indicates that digital transformation requires continuing strategic adjustment [2,7] and that the timing and coordination of strategic actions influence organizational adaptation [8,12]. Managers should therefore establish regular review cycles for digital initiatives and coordinate the timing, frequency, and sequence of major actions across functional units. Relevant indicators include the frequency of strategic adjustments, the continuity of digital initiatives, and the degree of coordination among related projects. Monitoring these indicators can help firms maintain a stable digital strategic rhythm while preserving enough flexibility to respond to technological and market change.

Second, managers should align network development with specific innovation objectives. Structural embeddedness improves access to diverse knowledge resources [14], whereas relational embeddedness facilitates trust-based knowledge exchange [15]. Firms pursuing exploratory innovation should therefore broaden their external connections, improve their network positions, and expand access to heterogeneous knowledge. Managers can monitor the number and diversity of external partners, the proportion of newly established network connections, and the share of new knowledge sources entering the firm. Firms pursuing incremental innovation, by contrast, should emphasize repeated interaction with key partners, continuity of cooperation, and efficient knowledge exchange. Relevant indicators include interaction frequency, cooperation duration, knowledge-sharing efficiency, and the stability of key partner relationships. These measures can help managers assess whether network development remains aligned with the firm’s innovation objectives.

Third, managers should establish regular digital sensemaking routines to improve how digital strategic actions are interpreted and communicated. Sensemaking helps organizations interpret digital change, develop shared understanding, and coordinate organizational responses [16,17]. Managers can convene regular cross-functional meetings to interpret technological developments, review market signals, and assess whether current digital initiatives remain aligned with strategic objectives. Structured reviews after major digital projects can also identify changes in technologies, partner requirements, and emerging network opportunities. These routines can strengthen shared understanding and help firms identify valuable external connections. Because relational embeddedness depends more heavily on repeated interaction, accumulated trust, and relationship history [15], managers should complement digital sensemaking with sustained relationship-building practices when deeper and more stable collaboration is required.

### 5.3 Limitations and future research

This study has three limitations. First, it examines HGFs in China; future research could investigate whether the relationships among digital strategic rhythm, network embeddedness, and innovation differ across institutional environments. Second, the cross-sectional design limits causal inference. Longitudinal studies could examine how digital strategic rhythm evolves and is associated with innovation over time. Third, this study focuses on digital sensemaking as a cognitive boundary condition. Future research could examine other contextual factors, such as environmental uncertainty and partner dynamics, that may influence the translation of strategic rhythm into network advantages.
